# Risk stratification of sudden cardiac death in Brugada syndrome: an updated review of literature

**DOI:** 10.1186/s43044-022-00267-9

**Published:** 2022-04-11

**Authors:** Charmake Darar, El-Azrak Mohammed, Boutaybi Mohammed, El Ouafi Noha, Bazid Zakaria

**Affiliations:** 1 Departement of Cardiology, Mohammed VI University Hospital, Faculty of Medicine and Pharmacy, Oujda/Mohammed I University, Oujda, Morocco; 2 Epidemiological Laboratory of Clinical Research and Public Health, Mohammed VI University Hospital, Oujda, Morocco

**Keywords:** Brugada syndrome, Sudden cardiac death, Risk stratification, Ventricular fibrillation, Channelopathy

## Abstract

Brugada syndrome is a rare but serious inherited heart disease that causes sudden cardiac death by polymorphic ventricular tachycardia or ventricular fibrillation. It is an autosomal dominant genetic disease that usually occurs in patients in their forties with a structurally normal heart. Electrically, it manifests by ST elevation segment ≥ 2 mm of at least one right precordial lead (*V*1 and/or *V*2). Stratification of sudden cardiac death in Brugada syndrome is not always easy and constitutes a real challenge for the practitioner. In this review, we will present the current state of knowledge for arrhythmic risk stratification and the prevention of sudden cardiac death that can result from this syndrome.

## Background

Brugada syndrome is one of the inherited heart conditions that are responsible for sudden cardiac death caused by sustained ventricular arrhythmias. Although there has been progress in the diagnosis and management of the cardiac channelopathies, the stratification of the risk of sudden cardiac death in Brugada syndrome is not always easy and still not well defined yet. It is from this perspective that this review is interested in providing current knowledge in terms of arrhythmic risk stratification in this condition. New studies are expected in the future to provide more convincing answers to this problem.

Brugada syndrome (BrS) is one of a particular group of inherited heart diseases called "channelopathies" which have a common denominator in the absence of underlying structural heart disease.

First identified in 1992 by brothers Pedro and Josep Brugada, this syndrome is characterized by the association of the right bundle branch block with ST segment elevation in the right precordium [1, 2].

With a prevalence of around 2 to 5/10,000, it exposes an increased risk of sudden cardiac death (SCD) by ventricular fibrillation, especially as it is symptomatic [2]. It is predominant in men with a sex ratio of 8/10 and is less common in children under 15 years and adults over 65 years [3].

It is a genetic disease with autosomal dominant transmission. The main gene involved in BrS is the SCN5A gene encodes the of the alpha-subunit sodium channel, although there is a panel of identified genes. These abnormalities are only found in 20 to 30% of cases. Due to its pronounced polymorphism, the genetics of BrS remain complex and some precaution should be exercised in genetic counseling [4–6]. More common in middle-aged men around the 4th-5th decade of life, BrS is only accompanied by symptoms (SCD, syncope, palpitations related to a supraventricular arrhythmia, etc.) only in a third of cases.

Asymptomatic patients are discovered either on the occasion of a fortuitous discovery of the electrical abnormalities on the ECG carried out for another reason, or during family screenings which are done more and more systematically.

In any case, the diagnosis of BrS is based on the conventional ECG recording of a type 1 pattern of BrS characterized by an elevation of the ST segment (≥ 2 mm) of at least one right precordial lead *V*1 and/or *V*2 followed by negatives *T* waves, whether spontaneous, or induced by a sodium blocker test [7–9]. In other words, only the presence of a type 1 pattern of Brugada can make the diagnosis of BrS [3, 10].

**Fig. 1:**
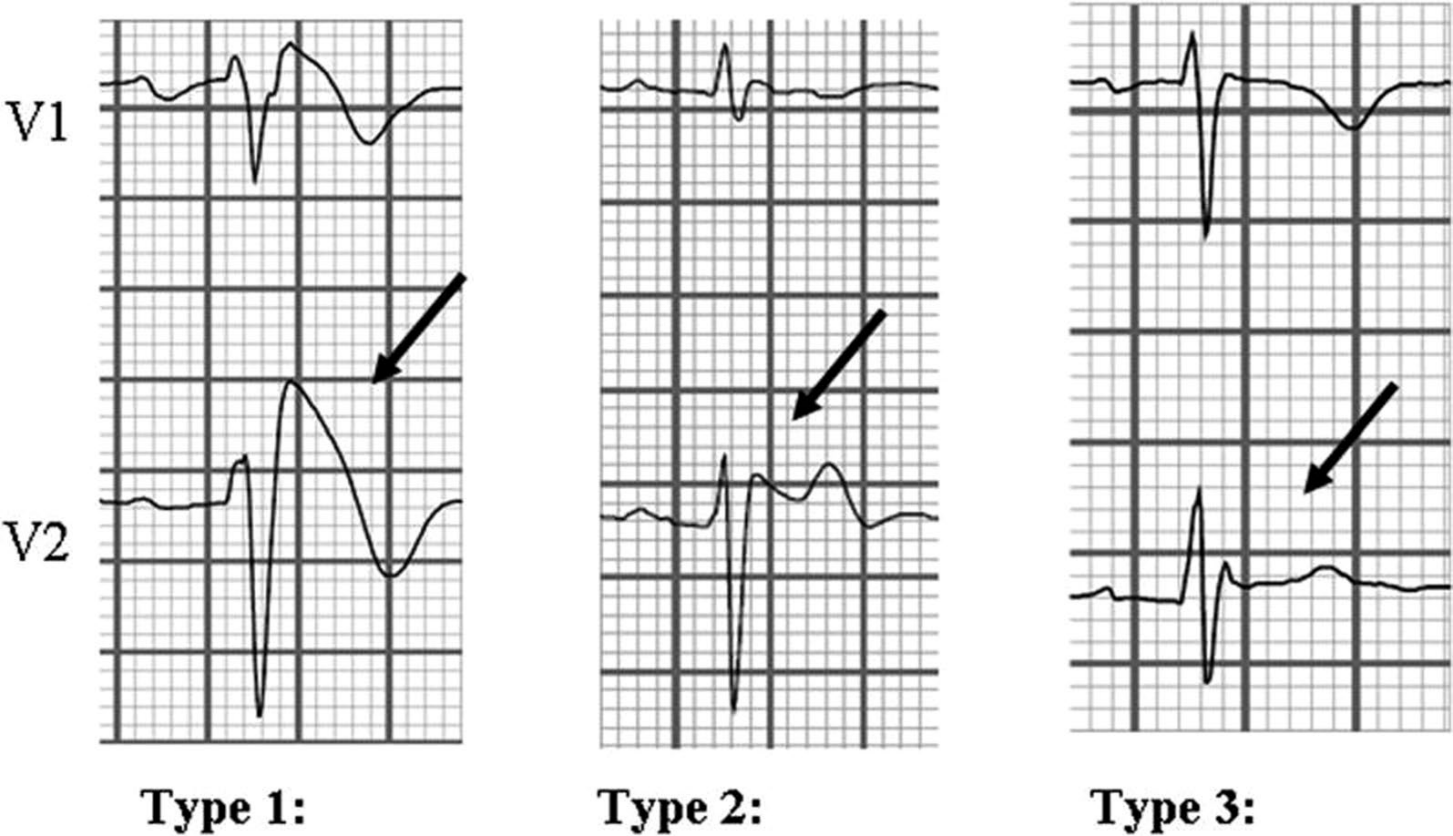
Illustration of 3 patterns of Brugada syndrome (published under CC BY-NC 3.0 license) [11]

Type 2 with biphasic *T* waves and type 3 with ST-elevation ≤ 1 mm are suggestive but insufficient to support the diagnosis of BrS. In these last two patterns, the diagnosis is retained if the administration of the sodium channel blocker (ajmaline or flecaine) converts a type 2 or type 3 pattern to a type 1 pattern [2, 11] (Fig. 1).

In order to increase the sensitivity of detection of an electrical aspect of Brugada, some authors have proposed to put the *V*1 and *V*2 leads higher on the thorax nearly of the 2nd intercostal space rather than the 4th intercostal space [11, 12].

However, a hypothetical relationship between the electrical aspect of Brugada and the risk of SCD was put forward in the late 1980s [7]. It was not until 1992 that the Brugada brothers described the syndrome as a definite clinical entity potentially responsible for SCD in patients with “structurally normal” heart [8].

However, the stratification of arrhythmic risk in patients with an electrical aspect of BrS is not at all simple and remains a matter of debate.

Indeed, this is a heterogeneous population of patients where some are at high risk of developing malignant ventricular arrhythmias, while others have a benign course. Some explanations for this fact, such as the dynamic character of the Brugada aspect, were hypothesized but remain insufficient. This is why risk stratification appears to be a “thorny” problem in clinical practice.

In this article, we will present the current knowledge on the risk stratification of SCD and its prevention in BrS.

BrS is responsible for 4% of all SCD and almost 20% of deaths in patients without structural heart disease [13].

Although not always easy, stratification of the risk of SCD in patients with BrS is required because it has an impact on subsequent management. This stratification takes into account several clinical parameters, electrical history, and programmed ventricular stimulation data.

### Recovered ventricular tachycardia (VT)/ventricular fibrillation (VF) or aborted sudden cardiac death

Any patient with severe ventricular arrhythmia (VT/VF) history or recovered SCD is considered high risk with an estimated recurrence rate of arrhythmic events of 48% over 10 years. In this group of patients, implantation of an implantable cardioverter-defibrillator (ICD) for secondary prevention is necessary without any additional assessment [14].

### Presumed arrhythmic syncope

The existence of syncope is a common manifestation found in up to 30% of patients with BrS [15, 16].

With an annual event rate of approximately 2%, only presumed arrhythmic syncope has been described as risk markers for SCD by ventricular arrhythmias (VT/VF) in this population [2, 17–20].

Other non-arrhythmic causes such as vasovagal syncope do not seem to confer an excess risk of death, although some studies have hypothesized that associated vagal hypertonia can precipitate the onset of VT/VF [21, 22].

Although not always easy, the differential diagnosis between these two syncope entities is of utmost importance because the implantation of an ICD is only recommended in patients with BrS and presumed arrhythmic syncope given the complications related to the device and its cost.

This distinction must be grounded on a good etiological approach based on some features.

In favor of the arrhythmic origin have been cited a family history of SCD, the existence of an underlying heart disease, the lack of prodromes and triggering factors, the “cookie-cutter” feature of the syncope, abnormalities of rhythm or conduction on the ECG, etc. [17, 23–25].

In favor of non-arrhythmic syncope, special situations have been reported such as prolonged standing, heat, a confined space, pain, stress, or emotion [26].

Palpitations, urinary incontinence, and traumatic injuries after the fall were common to both categories of syncope but were unspecific. Likewise, the occurrence of nocturnal syncope after awakening for micturition is not specific unlike the loss of consciousness during sleep, which is highly specific for arrhythmic syncope [27–29].

### The “thorny” question of asymptomatic patients

While sudden cardiac death is the most dramatic presentation, many patients with BrS are completely asymptomatic at the time of diagnosis. They represent 67 to 80% of several European registers.

Although their risk of death linked to a ventricular arrhythmia is lower compared to the symptomatic cases, this risk is not at all negligible; it is around 0.5% per year. Due to the uncommon and mildly severe presentation in women, the event rate was lower in the asymptomatic female population, around 0.27% per year [30, 31].

Stratification of the risk of sudden death in asymptomatic cases is essential as it is the basis of patients selection for ICD therapy. Nevertheless, it remains difficult to establish despite the many scientific advances.

So that further evaluation for risk factors is needed in order to classify patients as high and low risk for SCD [32].

### Age

It has long been known that the prevalence of BrS is lower in children and the elderly.

The absence of symptoms in children with BrS seems to be associated with a better prognosis, especially if they do not have a spontaneous type 1 pattern. However, their risk of SCD is not zero. For that, it is recommended to identify children of this high-risk age who may benefit from ICD therapy despite the absence of symptoms. This risk stratification by a pharmacological test or by an electrophysiological exploration must be individualized and interpreted with caution, taking into account the possible false negativity of a pharmacological test before the age of 15 years.

BrS in elderly patients has the particularity of being infrequent and of good prognostic value since according to some authors, the risk of SCD at this age of life is low or linked to other causes such as ischemic heart disease. In this case, the decision to implant an ICD must be made on an individual basis [33].

### The female gender

Long considered a “male” disease, BrS does not spare women [2, 30]. In the latter, it has the peculiarities of being more benign with less spontaneous type 1 pattern encountered, a lower annual event rate of around 0.27% and is most often asymptomatic. Nevertheless, they are unsafe to arrhythmic complications, and apart from a few ECG abnormalities such as atrial fibrillation (AF), prolonged PR interval, and a history of sinus dysfunction, reported to be related to the prognosis, there is a serious lack of the risk stratification in women [20, 34].

### Family history of sudden cardiac death

The role of a family history of SCD in BrS is controversial. Some report that this history, especially in first-degree relatives, was associated with other arrhythmic events, whereas in multivariate analysis, the link between the two facts was not significant [35].

### Genetic

As previously stated, a pathogenic mutation is only found in 20 to 30% of BrS.

The value of genetic data in stratifying the risk of sudden death in asymptomatic patients with BrS also remains controversial.

A possible relationship has been hypothesized between certain genetic mutations and the presence of symptoms or prolongation of the PR space. All of these data were considered statistically insignificant and have yet to be confirmed in larger studies [33].

### A spontaneous type 1 pattern of Brugada

The spontaneous type 1 pattern of Brugada is not commonly found in most asymptomatic patients, but its existence confers a risk three to four higher in asymptomatic patients [1, 36–41] justifying only careful monitoring and lifestyle modification [42]. In other words, the elevation of the ST segment characteristic of spontaneous type 1 Brugada pattern has a well-demonstrated prognostic value since patients with it have a worse prognosis, although type 1 induced by sodium channel blockers is not free from risk.

Spontaneous type 1 is not always easy to demonstrate since there are spontaneous fluctuations or secondary to taking drugs that influence variations in the ST segment [33].

Among the other ECG parameters that may increase the arrhythmic risk, there are the presence of fragmented QRS presents up to a third of asymptomatic patients with Brugada syndrome, atrial fibrillation [43, 44], and the existence of an electrical morphology of early repolarization in the inferior and lateral leads [45–47]. The association of fragmented QRS with the early repolarization aspect could confer with asymptomatic patients at very high risk [46, 48].

With its high negative predictive value close to 100%, the presence of an *S* wave of amplitude ≥ 0.1 mV (1 mm) and/or duration ≥ 0.04 s has been described as associated with a greater risk of VT and VF in patients with an aspect of Brugada. It is also observed the role of the alternating T waves and the AVR sign, but all these signs need to be confirmed in larger studies [49].

### The square of programmed ventricular stimulation

The place of electrophysiological stimulation (EPS) to assess the risk stratification of asymptomatic patients with BrS remains controversial and difficult to specify. Contrary to the first data, notably that of Brugada [50] which considered the EPS as a strong element of risk stratification, in multivariate analysis, the latter lost its statistical power in the subgroups of asymptomatic patients of the European Finger registry coordinated in 2010 by Probst [31].

Then the prospective American PRELUDE registry, which involved 308 patients, 80% of whom were men and in whom a programed stimulation was performed with an average follow-up of 34 months, had shown that the inducibility of VT/VF did not make it possible to identify high-risk patients [5].

A 20-year experience of Juan Sieira’s Italian team on 404 patients who received EPS and a mean follow-up of 74 months, the subgroup analysis of asymptomatic patients showed that the EPS had an interest in the prediction of the risk of rhythmic events, especially in patients with type 1 pattern of Brugada, whether spontaneous or caused by sodium channel blockers [51].

Due to its strong negative predictive value, negative EPS, i.e. without inducible VT/VF, gives asymptomatic Brugada patients a better prognosis. These patients only warrant close follow-up without the need for ICD implantation therapy.

Implantation ICD is only recommended in asymptomatic patients with BrS if they have a spontaneous type 1 pattern of Brugada and a positive EPS (recommendation IIA). The recommendation level drops to IIB if the type 1 pattern appears immediately after the drug challenge [52].

According to the latest consensus of 2015 on the management of BrS, 3 main criteria are retained to decide an ICD in asymptomatic patients: presence of a spontaneous type 1 pattern of Brugada or type 1 pattern induced by ajmaline or flecainide, a familial history of SCD and/or VT/VF inducible by EPS [53] (Table 1).

**Table 1:** Summary table of SCD risk stratification components and their references

Parameters	Influence on arrhythmic risk	References
Aborted sudden cardiac death	Increases the risk	[11, 14]
Presumed arrhythmic syncope	Increases the risk	[15–22]
Electrical pattern of BrS	Increases the risk	[37–43]
Age > 60 years old	Decreases the risk but needs to be confirmed	[33]
Female gender	Decreases the risk	[30, 34, 35]
VF inducibility in programmed ventricular stimulation	Increases the risk but there is a conflictual data	[5, 52, 53]
Sinus node dysfunction	Increases the risk but needs to be confirmed	[30, 33]
Fragmented QRS	Increases the risk but needs to be confirmed	[32, 45]
Early repolarization	Increases the risk but needs to be confirmed	[47–49]
Type 1 pattern in the inferior leads	Increases the risk but needs to be confirmed	[46, 48, 50]

## Conclusions

Brugada syndrome is a rare inherited heart disease associated with a significant risk of sudden cardiac death by ventricular fibrillation.

The diagnosis is based on the demonstration on a conventional ECG of a type 1 pattern, whether spontaneous, or induced by an ajmaline pharmacological test.

The arrhythmic risk stratification is high in the specific case of a spontaneous type 1 pattern, identification of suspicious symptoms, or an aborted sudden cardiac death history. In this group of patients, implantation of an ICD is essential.

Risk stratification of sudden death in asymptomatic patients remains a thorny question requiring discussion with competence centers and a personalized indication for ICD implantation.

## Data Availability

Not applicable.

## Abbreviations

BrS: Brugada syndrome
VT: Ventricular tachycardia
VF: Ventricular fibrillation
ICD: Implanted cardioverter defibrillator
EPS: Electrophysiological stimulation
SCD: Sudden cardiac death
